# Undiagnosed Diabetes: Identifying the Community Paths to Type 2 Diabetes Diagnostic Testing

**DOI:** 10.1093/geroni/igaa057.1211

**Published:** 2020-12-16

**Authors:** Sarah Chard, Laura Girling, Loren Henderson, J Kevin Eckert

**Affiliations:** 1 UMBC, Baltimore, Maryland, United States; 2 University of Maryland, Baltimore County, Baltimore, Maryland, United States

## Abstract

Over seven million U.S. adults are estimated to have undiagnosed diabetes and are at heightened risk of diabetes complications and poorer long-term glycemic control. Key to addressing undiagnosed diabetes is identifying how persons encounter diabetes testing in everyday life and the contextual factors that lead to consulting a health care provider. As part of the NIA-funded Subjective Experience of Diabetes Study we examined the pathways through which community-living African-American and non-Hispanic White older adults with type 2 diabetes (T2D) (N=75) received their T2D diagnosis. Systematic, thematic analyses using ATLAS.ti reveals three primary routes to diabetes diagnosis: diagnosis through continuity of primary care, diagnosis through happenstance testing, and diagnosis following the exacerbation of symptoms. While diagnosis as part of routine care was the least reported (N=13), participants’ accounts suggest diagnosis in primary care validates the patient-provider relationship and provides an occasion to calmly establish a treatment plan. More frequently, however, diagnosis occurs through fortuitous encounters with glucose tests, e.g., through work or community research projects (N=15) or after symptoms become alarming and disrupt daily life (N=47). Participants’ experiences in these latter two categories reveal the critical role of insurance and social prompts in the decision to consult a clinical provider regarding symptoms. At the same time, the abundance of over-the-counter therapies treating conditions commonly found early in the emergence of diabetes can delay clinical follow up. These findings highlight the importance of social prompts and community-based testing in the fight to reduce undiagnosed diabetes.

